# *N*-Methyl-*N*-Nitrosourea-Induced Photoreceptor Degeneration Is Inhibited by Nicotinamide via the Blockade of Upstream Events before the Phosphorylation of Signalling Proteins

**DOI:** 10.1155/2019/3238719

**Published:** 2019-04-23

**Authors:** Eriko Sugano, Kitako Tabata, Tsubasa Takezawa, Raki Shiraiwa, Hiroki Muraoka, Tomomi Metoki, Asaka Kudo, Yuki Iwama, Mitsuru Nakazawa, Hiroshi Tomita

**Affiliations:** ^1^Laboratory of Visual Neuroscience, Graduate Course in Biological Sciences, Iwate University Division of Science and Engineering, 4-3-5 Ueda, Morioka, Iwate 020-8551, Japan; ^2^Iwate University Division of Science and Engineering, 4-3-5 Ueda, Morioka, Iwate 020-8551, Japan; ^3^Department of Ophthalmology, Hirosaki University School of Medicine, 5 Zaifu-cho, Hirosaki, Aomori 036-8562, Japan; ^4^Clinical Research, Innovation and Education Center, Tohoku University Hospital, 1-1 Seiryo, Aoba, Sendai, Miyagi 980-8574, Japan

## Abstract

*N*-methyl-*N*-nitrosourea (MNU), a known carcinogen, is generally used in animal models to chemically induce photoreceptor degeneration. It has been reported that nicotinamide (NAM) exerts a protective effect on MNU-induced photoreceptor degeneration. We investigated the molecular mechanisms on MNU-induced photoreceptor degeneration. Intraperitoneal MNU injection (75 mg/kg) in rats induced selective photoreceptor degeneration in 7 days. NAM administration completely inhibited photoreceptor degeneration. Photoreceptor layer abnormality was observed within 6 hours after MNU injection, whereas it was restored in the NAM-treated retina, as detected by optical coherence tomography. One day following MNU administration, phosphorylation of the cell death-associated signalling proteins c-Jun N-terminal kinase (JNK) and p38 mitogen-activated protein kinase (p38) increased, while the apoptosis-related proteins, full-length poly(ADP-ribose) polymerase (PARP) and apoptosis-inducing factor (AIF), were depleted. These changes were not observed in the NAM-treated retinas. Cell survival signalling, such as extracellular signal-regulated kinase (ERK), Akt, and cAMP response element binding protein (CREB) phosphorylation, increased in the MNU- but not in the NAM-treated rat retinas. Increased phosphorylated ERK (p-ERK) levels were observed within 6 hours after MNU administration, suggestive of cell survival signalling activation. This did not occur in NAM-treated retinas. These results indicate that NAM regulates upstream cellular events prior to the activation of cell death-related signalling events, such as JNK and p38 phosphorylation.

## 1. Introduction

Photoreceptor degenerative diseases, such as retinitis pigmentosa (RP) and age-related macular degeneration (AMD), are a major cause of blindness. Although these two conditions present with similar pathological symptoms with respect to photoreceptor degeneration, they have different causative mechanisms. RP is a genetic disorder with various associated genetic mutations having been identified, mainly in relation to phototransduction pathways (https://sph.uth.edu/Retnet/home.htm). On the contrary, the development of age-related macular degeneration (AMD) has been mainly associated with various environmental factors, such as smoking, obesity, and vitamin deficiencies with only a few disease-associated mutations having been identified to date [1].

There are various types of photoreceptor degeneration animal models that simulate human pathology and represent important tools for investigating the molecular mechanisms behind RP and AMD and potential treatment targets. Rodent models of photoreceptor degeneration can be classified into two types: rat models, such as P23H [2], S334ter [3], and the one developed by the Royal College of Surgeons [4, 5], that harbour degenerative genetic mutations, and the models of photoreceptor degeneration that are induced by light [6, 7] or chemically [8–11]. Although various genetic mutations that lead to RP have been identified, they all eventually lead to photoreceptor cell apoptosis [12, 13]. Photoreceptor degeneration induced by light or chemicals is also associated with cellular apoptosis. Apoptosis is the final common pathway in the photoreceptor degeneration though some differences of molecular mechanisms involve in between gene- and light- or chemical-induced photoreceptor degenerations.

Nicotinamide (NAM), a water-soluble B-group vitamin (vitamin B3), exerts protective effects against light- [14, 15] and* N*-methyl-nitrosourea (MNU)-induced [16] photoreceptor cell apoptosis. A better understanding of the molecular mechanisms involved in the photoreceptor degeneration and NAM-mediated cellular protection will aid in the identification of successful therapeutic approaches against retinal degenerative diseases.

## 2. Materials and Methods

### 2.1. Animals

All animal experiments were conducted in accordance with the guidelines of the Animal Experiment Committee of Iwate University, Japan. Male Sprague-Dawley rats were obtained from CLEA Japan, Inc. (Tokyo, Japan). The rats were housed in a 12 h/12 h light/dark cycle with access to water ad libitum.

### 2.2. Antibodies

Antibodies against extracellular signal-regulated kinase (ERK; Cat No. 4695S), phosphorylated ERK (pERK; Cat No. 9101S), Akt (Cat No. 9272), pAkt (Cat No. 9271L), cAMP response element binding protein (CREB; Cat No. 9197S), and pCREB (Cat No. 9198S) were obtained from Cell Signalling Technology (Tokyo). The anti-glutamine synthetase (GS) antibody (Cat No. MAB302) was obtained from Millipore (Tokyo). The anti-c-Jun N-terminal kinase (JNK; Cat No. sc-571) and phosphorylated JNK (Cat No. sc-6254) were obtained from Santa Cruz Biotechnology. Secondary antibodies against rabbit (Cat No. S3731) and mouse immunoglobulin (Ig) G (IgG; Cat No. S3721) were obtained from Promega (Tokyo).

### 2.3. MNU and NAM Administration

Rats were administered a single intraperitoneal injection of MNU (Fujifilm Wako Pure Chemical Corp., Osaka, Japan) at a dose of 75 mg/kg body weight. The MNU solution was freshly prepared with sterile physiological saline containing 0.05% acetic acid immediately prior to use and stored at 4°C in the dark. NAM (Nacalai Tesque, Kyoto, Japan) was freshly dissolved in sterile physiological saline and subcutaneously injected at a dose of 1000 mg/kg, 30 minutes prior to MNU administration.

### 2.4. Histology

Seven days following MNU injection, rats were sacrificed using carbon dioxide, and eyes were enucleated. Eyes were immersed in 4% paraformaldehyde in phosphate buffered saline (PBS) overnight at 4°C and then embedded in paraffin. Serial sections (4 *μ*m) of the whole eye were cut sagittally through the cornea and parallel to the optic nerve, stained against haematoxylin and eosin, and visualised by microscopy (AxioImager A1; Carl Zeiss, Tokyo, Japan).

### 2.5. Optical Coherence Tomography (OCT)

Rats were anaesthetized by intramuscular injection of ketamine (75 mg/kg) and medetomidine (0.5 mg/kg) and their pupils were dilated with tropicamide (Midrin-P, Santen Co., Ltd., Osaka, Japan). The eye was placed under local anaesthesia using oxybuprocaine (Santen Co., Ltd., Osaka, Japan) and covered with a contact lens. Image acquisition of 1.1 mm length of the rat retina including the optic disk was performed using the line scan mode on an OCT imaging device equipped with a special ordered lens (RS-3000, NIDEK Co., Ltd., Aichi, Japan).

### 2.6. Western Blot Analysis

Western blot analysis was performed as previously described [17]. Briefly, the retinas were isolated from the eyes 1 and 3 days after MNU or MNU/NAM administration. Proteins were extracted from the samples with a lysis buffer containing 10 mM Tris-HCl (pH 7.5), 1% Triton X-100, 0.5% NP-40, 1 mM EDTA, 150 mM NaCl, 1x protease inhibitor cocktail (Thermo Scientific, Tokyo), and 1x Halt™ phosphatase inhibitor cocktail (Thermo Scientific, Tokyo). The lysate was quantified using a bicinchoninic acid assay (BCA) protein assay kit (Pierce, Rockford, IL). Thirty micrograms of protein was loaded for electrophoresis on 4–15 % Mini-PROTEIN TGX gels (Bio-Rad, Tokyo) and transferred onto a polyvinylidene difluoride (PVDF) membrane. After blocking with Block Ace (DS Pharma Biomedical, Osaka, Japan), the membrane was incubated with the appropriate primary antibody. Following washing, the membrane was incubated with the appropriate alkaline phosphatase-conjugated secondary antibody. Chemiluminescence detection (CDP-Star Detection Reagent; GE Healthcare, Tokyo) was performed according to the standard procedure. Band densities were measured using ImageQuant software (GE Healthcare, Tokyo) and the density of each band was normalised to that of *β*-actin.

### 2.7. Immunohistochemistry

Immunohistochemistry was performed on the retina 6 hours and 1 day after MNU or MNU/NAM administration. Paraffin-embedded sections were deparaffinized according to the standard procedure. The sections were treated in citrate buffer (pH 6.0) in a microwave for antigen retrieval. After blocking, the sections were incubated with anti-pERK (dilution 1:200) and GS antibodies as a marker of Müller cells or with a control solution of anti-rabbit and anti-mouse IgGs overnight at 4°C. The sections were then washed three times with PBS containing 0.01% Tween-20 and incubated with Alexa 488- or Alexa 594-conjugated anti-rabbit and anti-mouse IgG solution at room temperature for 1 h. Following washing, the sections were covered with mounting media including 4′,6-diamidino-2-phenylindole (DAPI; VECTASHIELD: Funakoshi, Tokyo).

*Statistical Analysis*. Statistical analysis was performed using GraphPad Prism 4 (GraphPad software, San Diego, CA). The statistical method used was Tukey's multiple comparison test.

## 3. Results

### 3.1. Histology

**Figure 1:**
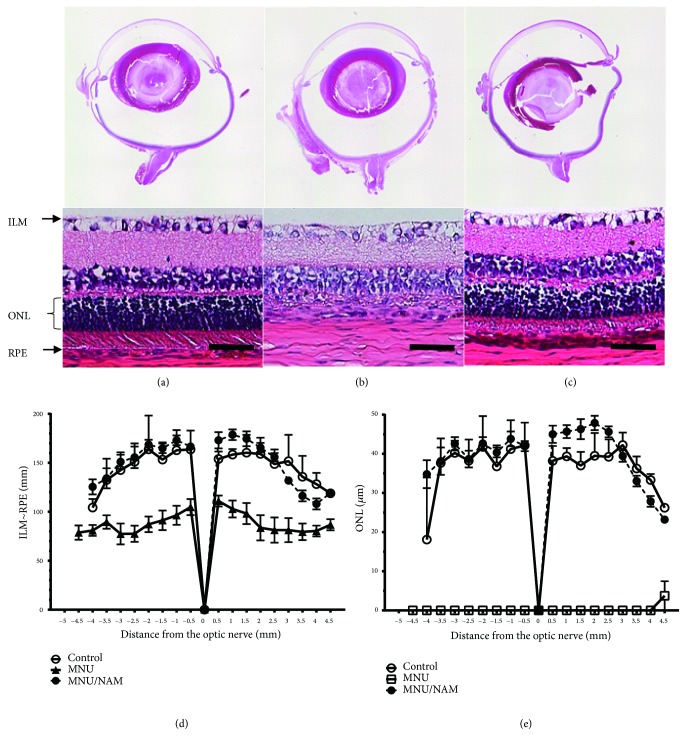
Histological retinal examination seven days after MNU or MNU/NAM administration. All retinal layers were clearly observed in control retinas (a). Photoreceptor cells were completely degenerated in the retinas of MNU injected rats (b), whereas they were rescued from the degeneration by subcutaneous NAM injection (c). Scale bar is indicative of 50 micrometres. The thickness of whole retina ((d) from ILM to RPE) and the ONL (e) were measured at 0.5 mm interval between the superior (+) and inferior (-) part. Data are shown as mean ± standard error (n = 4–8). MNU:* N*-methyl-*N*-nitrosourea; NAM: nicotinamide; OCT: optical coherence tomography; ONL: outer nuclear layer; PRE: retinal pigment epithelium; and ILM: internal limiting membrane.

Seven days following the intraperitoneal injection of MNU in rats, the outer nuclear layer (ONL) of the retina was not detectable (Figure 1(b)) and photoreceptor degeneration occurred in the whole retina (Figure 1(e)). The MNU-induced degeneration was localised to the ONL and did not affect any of the other retinal layers (Figure 1(d)). On the contrary, the retinal morphology was maintained in NAM-treated animals (Figures 1(c) and 1(e)), similar to that in controls (Figure 1(a)).

### 3.2. OCT

**Figure 2:**
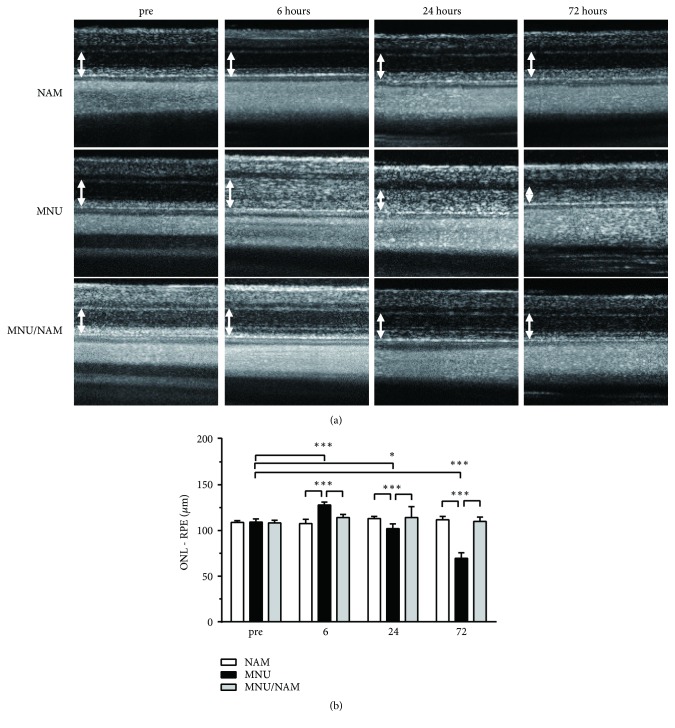
Photoreceptor degeneration induced by intraperitoneal MNU injection. OCT images were obtained from rats before and after administration of MNU, NAM, or MNU/NAM (a). The thickness of the photoreceptor layer (arrow) was measured (b). The photoreceptor layer (arrow) was present in the retina during MNU preadministration. The photoreceptor layer became thicker 6 hours after MNU injection and then gradually thinner at later time points. Data are shown as mean ± standard error (n = 4–16, *∗*, *∗∗∗*:* p* < 0.05, 0.001, Tukey's multiple comparison test). MNU:* N*-methyl-*N*-nitrosourea; NAM: nicotinamide; and OCT: optical coherence tomography.

The early phase of photoreceptor degeneration was evaluated by OCT (Figure 2(a)). The ONL was poorly marginated at 6 hours and 1 day after MNU administration and its thickness significantly increased at 6 hours, but decreased 1 day and 3 days after MNU administration (Figure 2(b)). This ONL thinning induced by MNU was completely blocked by NAM coadministration and the retinal architecture remained unchanged at all time points following NAM treatment.

### 3.3. Signalling Protein Expression

**Figure 3:**
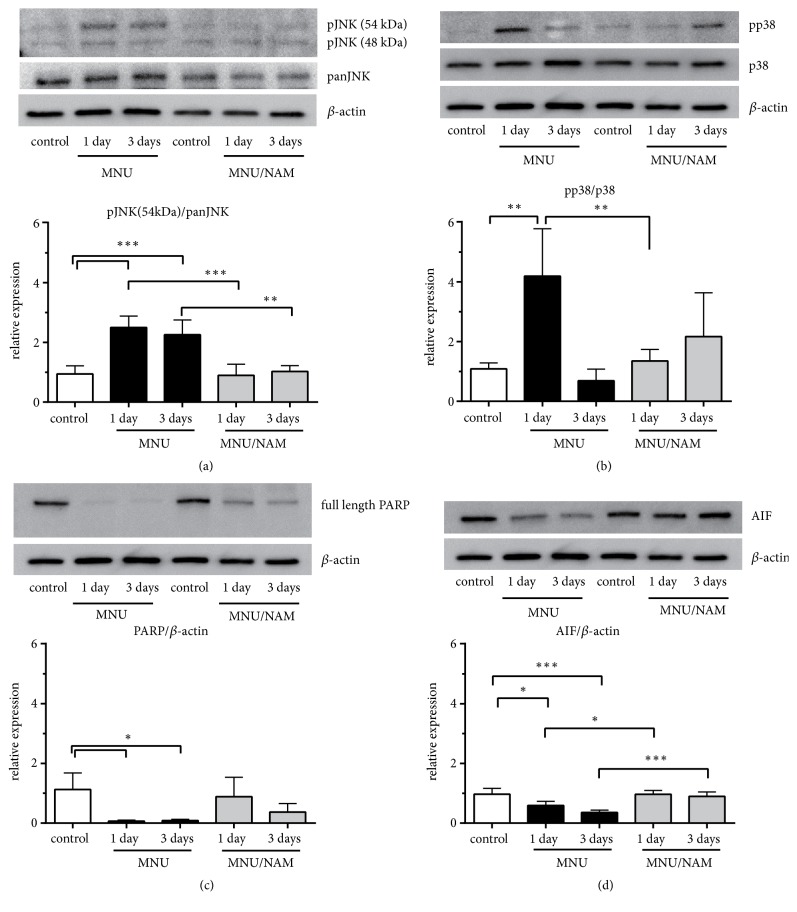
Effect of NAM on cell death related signalling proteins. Representative western blots of JNK (a), p38 (B), PARP (c), and AIF (d) are indicated in each densitometry analysis graph. Increased phosphorylated forms of JNK and p38 were observed in retinas 1 and 3 days and 1 day after MNU injection, respectively. The depletion of full-length PARP was observed in MNU-injected retinas. The expression level of AIF was decreased after MNU injection. Data are shown as mean ± standard deviation (n = 4, *∗*,*∗∗*, and *∗∗∗*:* p* < 0.05, 0.01, and 0.001, Tukey's multiple comparison test). AIF: apoptosis-inducing factor; JNK: c-Jun N-terminal kinase; MNU:* N*-methyl-*N*-nitrosourea; NAM: nicotinamide; PARP: poly(ADP-ribose) polymerase; and p38: p38 mitogen-activated protein kinase.

**Figure 4:**
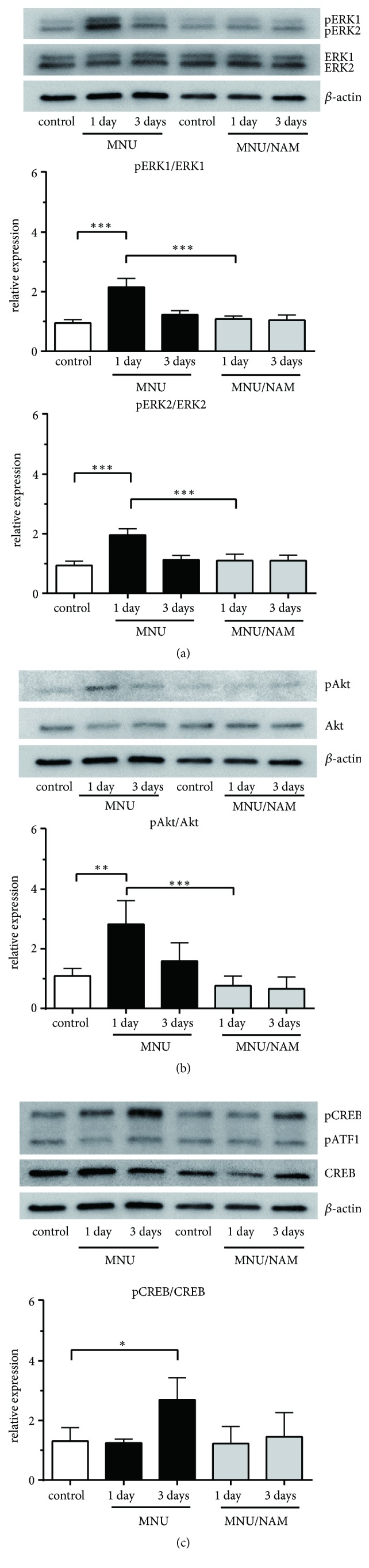
Effect of NAM on cellular stress-related proteins. Representative western blots of ERK (a), Akt (b), and CREB (c) are indicated in each densitometry analysis graph. Increased phosphorylated forms of pERK1, pERK2, and pAKT were observed at Day 1 after MNU administration. Thereafter, pCREB was also upregulated. Data are shown as mean ± standard deviation (n = 4, *∗*, *∗∗*, and *∗∗∗*:* p* < 0.05, 0.01, and 0.001, Tukey's multiple comparison test). CREB: cAMP response element binding protein; ERK: extracellular signal-regulated kinase; MNU:* N*-methyl-*N*-nitrosourea; and NAM: nicotinamide.

We sought to evaluate possible expression changes in cell death-related proteins. The phosphorylation of JNK (Figure 3(a); 54 kDa and 48 kDa) and p38 mitogen-activated protein kinase (p38; Figure 3(b)) was significantly increased 1 day after MNU administration. The increase in phosphorylated JNKs (pJNKs) was maintained for 3 days following MNU injection (Figure 3(a)). The expression levels of both poly(ADP-ribose) polymerase (PARP; Figure 3(c)) and the apoptosis-inducing factor (AIF; Figure 3(d)) were decreased after MNU injection. These changes were not observed in the retinas of the animals cotreated with NAM. Next, we examined the expression of various markers of cellular proliferation and survival. The levels of pERK (Figure 4(a)) and pAkt (Figure 4(b)) increased at 1 day, and the pCREB (Figure 4(c)) significantly increased after MNU injection. This MNU-induced protein phosphorylation was completely abolished by NAM coadministration (Figures 4(a), 4(b) and 4(c)).

### 3.4. Phospho-ERK and GS Immunohistochemistry

**Figure 5:**
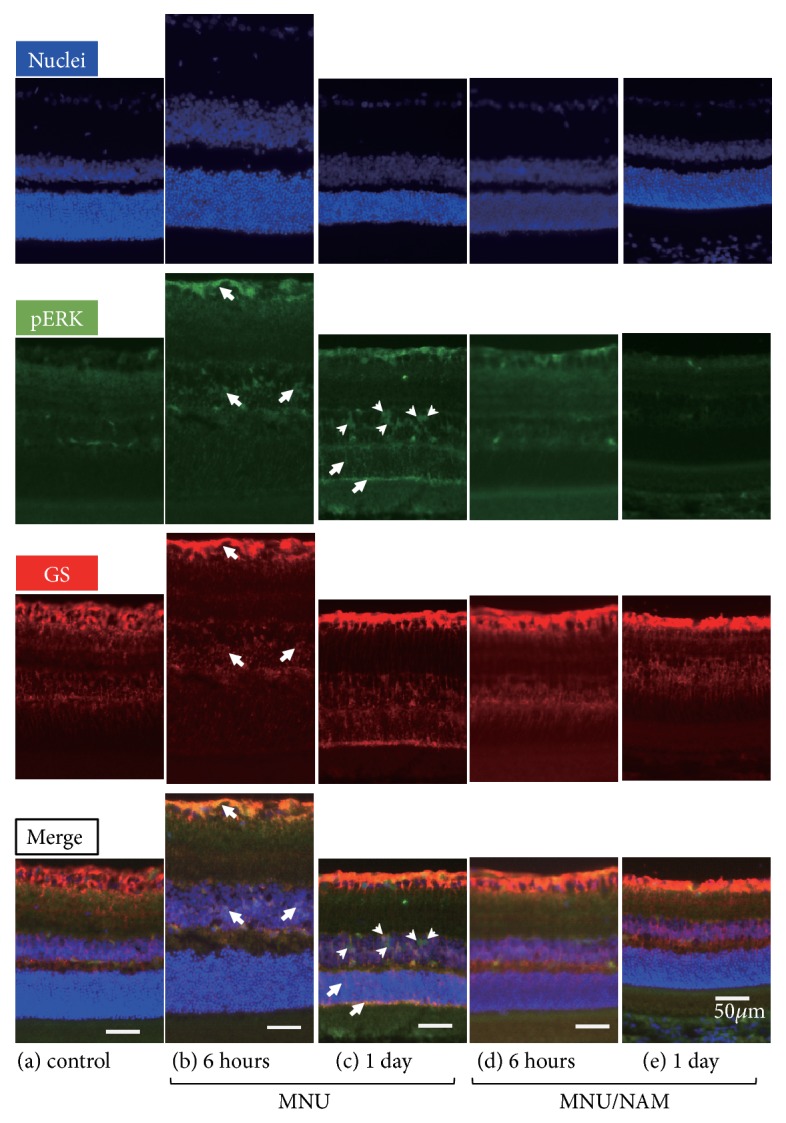
Retinal immunohistochemistry of pERK and GS after intraperitoneal MNU injection. Retinal cryosections from control (a), 6 hours (b, d) and 1 day (c, e) after MNU (b, c) or MNU/NAM (d, e) injection were immunostained against pERK and GS. The inner limiting membrane and the cell bodies of the inner nuclear layer were colabelled with pERK and GS antibodies 6 hours after MNU injection (arrows in (b)). One day after MNU injection, pERK immunoreactivity was observed in the outer nuclear layer and the outer limiting membrane (arrows in (c)). In addition, nuclear pERK staining was observed (arrowheads in (c)). The nuclei was stained with 4′,6-diamidino-2-phenylindole (DAPI) shown as blue. GS: glutamine synthetase; pERK: phosphorylated extracellular signal-regulated kinase; MNU:* N*-methyl-*N*-nitrosourea; and NAM: nicotinamide.

Phospho-ERK is a well-known marker of retinal stress with protective effects against retinal injuries [17–19]. We evaluated the early cellular responses following MNU administration. Phospho-ERK immunoreactivity (IR) was mainly observed in the inner nuclear layer and partly in the ganglion cell layer in control retinas (Figure 5(a)). However, six hours after MNU injection, pERK IR was mainly localised in the inner limiting membrane and the cell bodies of the inner nuclear layer (Figure 5(b)). Phospho-ERK IR was subsequently detected in the outer nuclear layer and the outer limiting membrane 1 day after MNU administration (Figure 5(c)), with predominant nuclear localisation. GS IR was consistently detected from the inner limiting membrane to the outer limiting membrane in control retinas. GS IR significantly increased 1 day after MNU injection in the outer nuclear layer (Figure 5(c)). When NAM was coadministered, retinal GS IR was slightly increased, as compared to controls, but no changes were observed in pERK levels (Figures 5(d) and 5(e)).

## 4. Discussion

MNU induced selective retinal photoreceptor degeneration in our rat model. Photoreceptor cell death was completely induced 7 days after MNU injection. OCT revealed photoreceptor layer degeneration as early as 6 hours after MNU injection, indicating the quick activation of the cell death signalling pathway (within 6 hours; Figure 2(a)). However, NAM cotreated retinas were indistinguishable from controls in OCT, as early as 6 hours following administration. We hypothesized that NAM regulates the early phase of photoreceptor degeneration to induce such protective responses against MNU-induced toxicity.

Previously, the JNK/stress-activated protein kinase (SAPK) signalling pathway has been associated with MNU-induced photoreceptor cell death [20] and is known to induce neuronal apoptosis [21, 22]. It has also been reported that the cell death pathway induced by the phosphorylation of p38 plays an important role in light-induced photoreceptor degeneration [23]. We detected MNU-mediated increases in pJNK and p-p38. NAM administration completely abolished these changes (Figures 3(a) and 3(b)) and blocked the MNU-induced photoreceptor degeneration (Figures 1(c), 1(d), and 1(e)). Nevertheless, there is little evidence supporting the direct inhibition of pJNKs or p-p38 by NAM. MNU is an alkylating agent that causes DNA damage [24]. PARP is a known immediate cellular response activator following alkylating agent-induced DNA damage [25]. We demonstrated that MNU leads to the depletion of full-length PARP within a day after its administration (Figure 3(c)) and a subsequent decrease in AIF expression (Figure 3(d)). Uehara N. et al. previously showed the increased PARP in the photoreceptor cells with the same model [20]. Our results seemed to be controversial. However, the MNU concentration used in this study was higher than that they used, indicating that more severe retinal damage was induced in our model. The PARP would be cleaved by the MNU administration, and PARP and AIF were possibly translocated into the nucleus. Subsequently, the full-length PARP and AIF levels in the cellular component were decreased. These results, at least, indicate that the PARP/AIF-associated cell death pathway [26] is involved in MNU-induced photoreceptor degeneration. The nuclear translocation of PARP leads to the depletion of nicotinamide adenine dinucleotide (NAD^+^) and a decrease in cellular ATP levels, which in turn induce cell death [27]. Taking all these into consideration, it is likely that NAM acts as a NAD^+^ supplier or a PARP inhibitor as NAM is a precursor for NAD^+^, a known PARP inhibitor by pretreatment of NAM [28].

To further explore the effects of NAM on cellular signalling, pERK [18], pAkt [29], and pCREB [30] were evaluated with respect to their potential protective effects against retinal injuries. NAM administration did not affect the phosphorylation levels of these signalling proteins (Figures 4(a), 4(b) and 4(c)). On the contrary, MNU administration led to a significant increase in pERK and pAkt, as well as pCREB at later time points. Likewise, we have previously reported that Akt also contributes in photoreceptor survival and maintenance in light-induced photoreceptor degeneration [31]. Increased pERK IR was mainly observed in Müller cells, as early as 6 hours after MNU administration. It has been reported that the phosphorylation of signal transducer and activator of transcription 3 (STAT3) and ERK in Müller cells plays an important role in ciliary neurotrophic factor (CNTF)-mediated photoreceptor rescue in the retinal degeneration [32]. The observed MNU-mediated pERK increase in Müller cells might reflect an intrinsic retinal protection mechanism against injuries.

In this study, we evaluated potential changes in the expression patterns of various signalling proteins following MNU administration. The expression levels of all signalling proteins evaluated in this study were altered within one day after MNU injection. The immunohistochemical evaluation of pERK revealed the activation of self-protective retinal responses within 6 hours of MNU injection, which was not the case in the NAM cotreated retinas.

## 5. Conclusions

NAM is likely to regulate upstream signalling pathways, such as NAD^+^ consumption or PARP inhibition, to induce retinal protection against photoreceptor degeneration prior to the phosphorylation of signalling proteins that occurs in later stages. The results of this study provide a better understanding of the molecular mechanisms underlying retinal degeneration and aid in the identification of novel therapeutic targets against photoreceptor degenerations.

## Data Availability

The data used to support the findings of this study are available from the corresponding author upon request.
